# Lessons Learned From a Low-Income Country to Address Mental Health Needs During COVID-19

**DOI:** 10.3389/fpsyt.2021.576352

**Published:** 2021-06-10

**Authors:** Judite Blanc, Azizi Seixas, Elizabeth Farrah Louis, Donaldson Fadael Conserve, Georges Casimir, Girardin Jean-Louis

**Affiliations:** ^1^Department of Population Health, New York University, New York, NY, United States; ^2^Fogarty International Center (FIC), Bethesda, MD, United States; ^3^University of South Carolina Lancaster, Lancaster, CA, United States; ^4^State University of New York Downstate Medical Center, Brooklyn, NY, United States

**Keywords:** 2019 coronavirus disease, Haiti 2010 earthquake, psychological first aid, war mentality, low and middle income country

## Introduction

As of April 2021, more than 130 million Coronavirus-2019 (COVID-19) confirmed cases and 2 million deaths were reported in 223 countries, territories or regions (1). The devastation caused by the global health crisis surpassed that which was caused by the severe acute respiratory syndrome (SARS) epidemic and the Ebola outbreak. Evidence shows COVID-19 is associated with high mortality, transmission rate, and strict biosecurity restriction. COVID-19 has affected many segments of society such as neighborhood quarantines, nighttime curfews, and states of emergency imposed worldwide. World economies have declined, borders and markets closed, trade and agricultural production have faltered, creating cascades of human and material losses. Psychological stress above and beyond the fear-provoking consequences of the disease itself.

A systematic review of cross-sectional studies published during the first 5 months of 2020 revealed an alarming rate of symptoms of anxiety (6.33 to 50.9%), depression (14.6 to 48.3%), posttraumatic stress disorder (7 to 53.8%), psychological distress (34.43 to 38%), and stress (8.1 to 81.9%) in the global population including in high and low- and middle-income countries such as the US, Denmark, Italy, Spain, China, Iran, Turkey, and Nepal. These studies also showed that the female gender, being$$ less than ≤40 years, a diagnostic of chronic/psychiatric ailments, unemployment, student status, and frequent exposure to social media/COVID-19 related news were risk factors for adverse mental health outcomes (2).

In light of these current and unimaginable realities of a global panic and trauma, it is thus incumbent on all of us to protect against, manage, and rebound from COVID-19. However, since the world has not dealt with the scope and magnitude of such a global disaster in a century (Spanish Flu being the last major pandemic), we were unprepared administratively and psychologically. We remain ill-equipped to handle the demands of COVID-19. To deal with this pandemic, some countries, such as the United States, have invoked the spirit of war to cope administratively and psychologically with the COVID pandemic (3). A war mentality may have psychological benefits in some instances, such as the appropriate lauding of healthcare and frontline workers as heroes and the sense of being part of a community fighting against a common enemy—COVID-19. Although a war mentality may positively affect the administrative response to fighting COVID-19 by providing a more robust supply chain management of personal protective equipment (PPE), it presents some drawbacks. A war mentality may not be the best approach to address and cope with COVID-19 pandemic as it will likely induce a constant state of fear, alarm, victimhood syndrome, and xenophobia. A war mentality may not be the best approach to cope with a pandemic, as it may induce victimhood syndrome where individuals protest against harsh public health policies such as shelter-in-place and physical distancing. The war mentality has to lead to higher levels of xenophobia against people of Asian descent, as seen in the horrific spate of recent attacks against Asian Americans in the United States. The use of “othering” caused by COVID-19 is a maladaptive coping strategy where foreigners are seen as scary and used as the scapegoats (e.g., the constant prejudicial undertones of COVID-19 being a Chinese virus) (4). All of these approaches are antithetical to the appropriate prescription and remedy of surviving a disaster.

## The Case for Psychological First Aid and Related Approaches During COVID-19

We argue that instead of a war mentality, a global natural disaster mentality, one rooted in psychological first aid framework and other related approaches such as psychoeducation, and psychosocial support is needed (5, 6). It will help us address the administrative and psychological needs to survive COVID-19. According to the Inter-Agency Standing Committee, common reactions during an outbreak may include avoidance of health facilities, fear of being infected, feeling powerless to protect oneself and others, anxiety, worry, loneliness with self-isolation, depression, triggers of previous stressors. One such stressor that could be particular for this outbreak is the risk of getting infected or infecting others since many guidelines are not being followed, mistaking other health problems with symptoms of COVID-19, and uncertainty about the outcomes of the pandemic (7). Given the prolonged uncertainty of the COVID-19 pandemic, one psychological model, psychological first aid could be helpful to recognize the potential mental health distress and stress that affected populations are facing (8).

The PFA is a novel intervention that respects the dignity, culture and capacities of others (9). According to Shultz and Forbes (2014). PFA is characterized by five key elements which are: safety, calming, connectedness, self-efficacy, and hope. In other terms, PFA is a human-centered and culturally-tailored early intervention that includes a provision of information, comfort, emotional care, and instrumental support to individuals exposed to acute stress. As a frontline intervention, PFA is not meant to be delivered by mental health professionals. The goal is to habilitate an array of lay workers, ranging from professional disaster responders (emergency services personnel, medical emergency teams) to teachers and clergy to administer it. PFA frameworks are currently largely accessible and available in multiple languages. Education on PFA is offered through a variety of live, online, mobile, and mediated training modalities (6).

In the following section, we will summarize our observation of how PFA and other related psychological frameworks were disseminated and integrated with the reinforcement of the Haitian mental health system; and into non-governmental organizations (NGO's) interventions in the aftermath of one of the most devastating disaster in Haitian history (8, 9), to address administrative and psychological needs of the country, and argue for the adoption of PFA for COVID-19.

## Learning From Haiti'S Psychological First Aid and Mental Health Strategy 2010 Earthquake

Haiti's response post-2010 earthquake paradigmatically changed how the country deals with natural disasters, specifically its public health response. Prior to the 2010 earthquake, there was no cogent mental health policy by the *Ministere de la Santé Publique et de la population* (Ministry of Health) *(MSPP)*, despite several attempts to create one by Bijoux (10). The undervalued priority of mental health issues in Haiti may explain the delayed creation and implementation of a mental health policy. Prior to the disaster, there was no systematic planning for services. The number of mental health professionals was very limited. In 2003, a joint Pan American Health Organization (PAHO) and WHO report identified ten psychiatrists and nine psychiatric nurses working in the public and private sectors in Haiti. In addition, these professionals work mostly in Port-au-Prince, where individuals have to travel from the countryside to receive mental health services. There is a psychiatric hospital in Port-au-Prince and another one about 22 miles from the capital of Port-au-Prince. In the second largest public hospital in the country, Hospital of Justinien in Cap-Haitien (northern side), with limited monthly psychiatric services (10). In 2011, in the field of mental healthcare services the WHO report identified:

- 27 psychiatrists (0.28 psychiatrist per 100.000 inhabitants)
- 14 physicians with no specialization in psychiatry (0.14 per 100.000 inhabitants)
- 36 nurses per 100.000 inhabitants
- 194 psychologists, i-e two per 100.000 inhabitants
- 82 social workers, i-e 0.86 per 100.000 inhabitants
- 1 occupational therapist
- 1 neurologist
- 2 speech therapists.

## Mental Health in the Aftermath of the HAITI 2010 Earthquake

Weeks after the earthquake, there was a mobilization of Haitian psychologists toward the implementation of a national mental health policy in the country. Several meetings were held from March 2010 to December 2010 to plan and implement the formation of l'Association Haitienne de Psychologie (AHPsy) (Haitian Psychological Association). The newly formed association aimed to partner with other stakeholders in defining a national plan for mental health. As a result, AHPsy held its first conference in June 2011. A plethora of local and international psychologists, psychiatrists, social workers, nurses, spiritual healers, instructors, and educators participated in that conference and shared their data, findings and knowledge on psychological trauma and other related issues (11). Three years later in 2014, a 60-pages document entitled “Mental Health Component” was published by the MSPP (12).

## Training of Human Resources in Psychological First Aid and Other Related Approaches

In the aftermath of the earthquake, Haiti became the new El Dorado as non-governmental organizations and international organizations (IOs) entered the country to provide emergency care and “psychological first aid” to amputees in a state of posttraumatic shock, and those with other physical injuries (9). Multiple training sessions, workshops and conferences between Haitian and foreign psychiatrists, psychologists and social workers were organized in Haiti and abroad.

## Implementation of Ideo and Uramel and First Psychological Trauma Center in Haiti

L'Institut de Development Personnel et Organisationnel- (IDEO) (*Institute for Personal and Institutional Development*) and the Unite de Recherche et d'Action Medico-Legale-URAMEL (*Unit of Research and Medico-Legal Action*) partnered to offer psychological first aid training and trauma-focused care before the 2010 earthquake. In the aftermath of the earthquake, they received international funding from “Terre des Hommes and Trauma Aid Germany” to launch Haiti's first comprehensive Psychological Trauma Center (Center de Psychotrauma) in Port-au-Prince. According to an interview with the Haitian online press agency *Alterpresse* in January 2014, URAMEL's head reported having conducted more than 17,000 therapy sessions for 3,869 people (13). The Report published in 2016 by IDEO (14) indicated that from December 2011 to April 2015, children's mental health and development were largely taken into account in their overall program.

## Integrative Approaches of PFA

### *Plas Timoun* (Children's Corner)

The First Lady of the Republic of Haiti, Elizabeth Préval, initiated a project called *Plas Timoun*, to help children cope with stressful events such as grievance, loss of homes, and relocation to temporary shelters, etc. A group of six buses were parked in the courtyard of the Haitian Art Museum in “Champ de Mars,” the largest public square in the capital at that time transformed into a vast temporary accommodation camp. In Champs de Mars, children benefited from eight workshops: painting, theater, reading, games, music, pottery, sport and socio-educational activities^1^.

### The Response of the Haitian Church With Catholic Relief Support

As a result of the support provided by Secours Catholique (*Catholic Relief*), a non-profit catholic association that indicated having provided psychological assistance to more than 15,000 children since 2012, the “Cellule D'Aide Psychologique” (Psychological Assistance Unit) (CAP-CHR) was implemented. In an attempt to respond adequately to the numerous requests for psychological services from the community, following January 2010, the Haitian Conference of Religious (CHR), through this structure, the CAP was initially created in support of religious structures. The CAP is a group of Christians with skills in psychology but also laypersons sharing the same Christian values. In May 2011, the association CAP-CHR was recognized by the Ministry of Social Affairs of Haiti. The purpose of the CAP-CHR was to mobilize and train teams of undergraduates in psychology to work with families, throughout schools and provide them with tools for their psychological reconstruction.

Eleven years after the most devastating natural catastrophe in Haiti's history, the memories and the scars of the earthquake are alive in the Haitian collective psyche. And Haitian mental health professionals unanimously acknowledged that the disaster shed light on the importance of mental health for disaster-preparedness initiatives in this disaster-prone island. From this psychological standpoint, what lessons could high-income countries learn from the Haitian experience with the 2010 earthquake? We recommend avoiding the spirit of war which was adopted by some world leaders as a coping strategy to COVID-19, rather in Table 1, we propose a roadmap toward the dissemination of culturally-tailored psychological first aid and other related approaches to address mental health outcomes of the pandemic.

**Table 1:** A roadmap toward the dissemination of culturally tailored psychological first aid frameworks during Covid-19 pandemic.

1. Dissemination of Psychological First Aid Guide for lay providers, ranging from professional disaster responders (emergency services personnel, medical emergency teams) to teachers and clergy.
2. Require that all schools and institutions of higher education include a mandatory psychological first aid module for all staff members and develop a mechanism to refer at-risk youth to mental health programs.
3. Implement free mental health programs for the most vulnerable groups such as essential workers (health-care and hospital personnel, public transportation, delivery and grocery workers); COVID-19 survivors (particularly those who presented with severe forms of the disease); the elderly, families who lost loved ones and those who lost their jobs due to the pandemic.
4. Provide tax incentives for the implementation of employer-sponsored wellness programs that promote mental health in at-risk communities.
5. Establish helplines with a diverse staff that mirrors that of the community and that require staff in the helpline to practice cultural humility.
6. Allocate funding to advance studies on mental health, psychological resilience to the COVID-19 pandemic.

## Conclusion

In summary, 10 years after the Haiti 2010 earthquake, great progress has been made in public policies, programs and the provision of services in the field of mental health. As a result of such progress, in April 2020, to ensure that psychological care became more accessible, AHPsy launched a *helpline*. For nearly 3 weeks, as COVID-19 made waves in the country, attendants received calls from dozens of Haitians in need of professional psychological support. According to the head of this service, the former President of AHPsy, “Although the service was not created specifically for COVID-19, it was set up in the midst of a coronavirus pandemic. Surprisingly, we are receiving very few cases of psychological distress in connection to the COVID-19 pandemic,” says Ronald Jean Jacques (15). Could Haiti's long history of coping with historical traumas and natural disaster a buffer against adverse mental health outcomes during COVID-19? One year since the first case of COVID-19 has been reported in Haiti, at the end of March 2021, Haiti was among the countries with the least COVID-19 confirmed cases (<13,000) and death (<300) (16). Certainly, some could argue that, this is due to a lack of testing, limited tourist activities and commercial relationships with the outside world. For historical and socio-economic reasons, global health authorities were anxious about the impact of COVID-19 on the first Black republic along with other low and middle-income countries (LMICs). As of April 2021, surprisingly, according to the Haitian Ministry of Health, Haiti is coping well with the COVID-19 pandemic.

## Author Contributions

All authors listed have made a substantial, direct and intellectual contribution to the work, and approved it for publication.

## Conflict of Interest

The authors declare that the research was conducted in the absence of any commercial or financial relationships that could be construed as a potential conflict of interest.

## Footnotes

^1^https://africa.si.edu/exhibits/haiti/plas.html.

## References

[1] WHO. WHO Coronavirus (COVID-19) Dashboard. COVID19. WHO (2021). Available online at: https://covid19.who.int/

[2] Xionga J Lipsitzc O Nasric F Luic LMW Gillc H Phanc L. Impact of COVID-19 pandemic on mental health in the general population: a systematic review. J Affect Disord. (2020) 277:55–64. 10.1016/j.jad.2020.08.001 32799105 PMC7413844

[3] Frieden T. Former CDC Director: There's a Long War Ahead and Our Covid-19 Response Must Adapt. CNN (2020). Available online at: https://www.cnn.com/2020/03/20/health/coronavirus-response-must-adapt-frieden-analysis/index.html

[4] Kuo L. Trump sparks anger by calling coronavirus the 'Chinese virus' [Online]. The Guardian. (2020, March 17). Available online at: https://www.theguardian.com/world/2020/mar/17/trump-calls-covid-19-the-chinese-virus-as-rift-with-coronavirus-beijing-escalates

[5] WHO. Mental Health Considerations during COVID-19 Outbreak [Online] (2020). Available online at: https://pscentre.org/?resource=9031

[6] WHO. Psychological First Aid: Guide for Field Workers Guide for Field Workers. WHO (2011).

[7] Pfefferbaum B North C. Mental Health and the Covid-19 Pandemic. N Engl J Med. (2020) 383:510–2. 10.1056/NEJMp2008017 32283003

[8] Shultz JM Forbes D. Psychological First Aid: Rapid proliferation and the search for evidence. Disaster Health. (2014) 2:3–12. 10.4161/dish.26006 28228996 PMC5314921

[9] Schafer A Snider L van Ommeren M. Psychological first aid pilot: Haiti emergency response. Intervention. (2021) 8:245–54. 10.1097/WTF.0b013e32834134cb

[10] OMS. Le Systeme de Sante Mentale en Haiti. Haiti: OMS (2011).

[11] Nicolas G Jean-Jacques R Wheatley A. Mental health counseling in haiti: historical overview, current status, and plans for the future. J. Black Psychol. (2012) 38:509–19. 10.1177/0095798412443162

[12] Ministère de la sante publique et de la population. Composante de Santé Mentale de la Politique Nationale de Sante. Port-au-Prince: Ministère de la sante publique et de la population (2014).

[13] Bruno E. Haïti-Séisme/4 ans : Faire le deuil des proches disparus, un cap difficile [Online]. Alterpresse (2014). Available online at: https://www.alterpresse.org/spip.php?article15771.

[14] IDEO. IDEO Presentation [Online]. Internal Report. Trauma Ressources International (2016). Available online at: https://traumaresourcesinternational.org/wpcontent/uploads/2016/10/Foundatino-IDEO-Pr%c3%a9sentation.pdf.

[15] Francisque J. “Tu es folle!”, “Un homme doit gérer ses problèmes seul”…visiter un psy reste mal vu en Haïti [Online]. Ayibopost (2020). Available online at: https://ayibopost.com/tu-es-folle-un-homme-doit-gerer-ses-problemes-seulsvisiter-un-psy-reste-mal-vu-en-haiti/

[16] Ministere de la Sante Publique et de la Population. COVID-19- Siyasyon Maladi a Ayiti [Online] (2021). Available online at: www.mspp.gouv.ht

